# Tuberculosis morbidity and mortality during the COVID-19 pandemic: a life-threatening complex challenge

**DOI:** 10.3389/fcimb.2024.1423081

**Published:** 2024-10-01

**Authors:** Alissar Al Khatib, Salwa Hassanein, Mohammed Almari, Mohamad Koubar, Suha Fakhreddine

**Affiliations:** ^1^ Department of Nursing, Faculty of Health Sciences, Almoosa College, Al Ahsa, Saudi Arabia; ^2^ Department of Community Health Nursing, Cairo University, Cairo, Egypt; ^3^ Department of Laboratory Sciences, Faculty of Public Health, Lebanese University, Beirut, Lebanon; ^4^ Department of Microbiology, Lebanese Food, Drugs and Chemicals Administration, Beirut, Lebanon; ^5^ Department of Infectious Diseases, Saint-Georges Hospital, Hadat, Lebanon

**Keywords:** tuberculosis, COVID - 19, morbidity, mortality, complex challenge

## Background

Tuberculosis (TB) is a communicable disease caused by the infectious agent *Mycobacterium tuberculosis* (WHO, 2019a). Tuberculosis is considered one of the major causes of global deaths after COVID-19 (WHO, 2023). Globally, about one-quarter of the population has been infected with TB, however only 5-15% may develop tuberculosis during their lifetime with an increase in the incidence rate in immunosuppressed people, particularly HIV-positive patients. Yearly, among the total number of people developing TB, around 90% are adults with more cases among men than women (Emery et al., 2021). Moreover, the World Health Organization (WHO) declared that among 26 high burden countries2 the treatment success rate was slightly higher in 2021 among females than males to achieve 90% and 88% respectively (WHO, 2023).

## Global tuberculosis burden

Worldwide, an estimated 10.6 million people (95% UI: 9.9–11.4 million) fell ill with TB worldwide in 2022, up from 10.3 million (95% UI: 9.6–11.0 million) in 2021 and 10.0 million (95% UI: 9.4–10.7 million) in 2020, Therefore, the scenario of downward trend in pre-pandemic may happen in 2024. On the other hand, African Region accounted for (68%) of global TB deaths among people with HIV (WHO, 2023). Studies have shown that adherence to an anti-TB drug regimen decreases the rate of death from 50% to 15%. Therefore, a regimen of 6 months was highly recommended by World Health Organization (WHO) guidelines (WHO, 2023). Although death is preventable by successful treatment, a great number of TB survivors experience post-tuberculosis conditions, with an increase of long-term disability evidence in this population (Nightingale et al., 2023). The burden of TB disease decreased in some countries to reach fewer than 1 death per 100,000 population and less than 10 cases per year (Global Burden Disease Compare (GBD), 2019; World Health Organization (WHO), 2019b). Accordingly, a reduction of TB incidence of 11% was recorded between 2015 and 2020 which is close to the 20% reduction planned by WHO as the end TB strategy milestone between 2015-2020 (WHO, 2021). Remarkably, the COVID-19 pandemic had an obvious impact on the TB burden, where it affected the number of newly reported cases of TB, besides the global drop between 2019 and 2020 in the percentage of TB patients receiving TB treatment, especially those covering the multi-drug resistant strains (WHO, 2023).

## Tuberculosis risk factors and health impact

Undernourishment, HIV infection, Alcohol Use Disorders (AUD), smoking, and diabetes are the top five TB risk factors (WHO, 2023). In addition to these health-related risk factors, socio-economic conditions increase people’s susceptibility for developing TB infection during their lifetime. Worldwide in 2021, TB incidence cases of 2.2 million, 0.86 million, 0.74 million, 0.63 million, and 0.37 million were attributed to the previously listed health-related risk factors respectively (WHO, 2021). Accordingly, Human immunodeficiency virus (HIV) ranks in second place after TB as a leading cause of death from a single infectious agent. Moreover, HIV-positive patients are 30 times more susceptible to developing active TB than HIV-negative ones, if left untreated the mortality is close to 100% (van Woudenbergh et al., 2020). Insulin dependence represents an additional risk factor for developing TB among diabetic patients, where the risk increases by two-fold in high insulin treatment dependence (Silva et al., 2018). Remarkably, the major impact of smoking in terms of public health issues is increasing susceptibility to infection with *Mycobacterium tuberculosis*, since the majority of tobacco consumers live in developing countries under unhealthy conditions, when it comes to mortality, the rate is nine times greater for smokers than non-smokers (Altet et al., 2017). The association of TB-alcohol has long been known; studies have shown that alcohol use is responsible for around 10% of TB cases worldwide (Silva et al., 2018). Based on what we have discussed previously about the complex association between undernutrition and TB, undernourishment is the most important risk factor to know where and how to address. Therefore, a guidance document on undernutrition in TB patients was elaborated by WHO to prioritize areas that need close monitoring for undernutrition in TB, since decreasing TB risk in a community is tackled by improving its nutritional conditions (Carwile et al., 2022). Since addressing malnutrition in TB patients is challenging, Damji and his collogues proved that patients undergoing TB treatment demonstrated the difficulty in appropriately meeting nutritional needs, even when providing nutritional support (Damji et al., 2022).

Unfortunately, some countries with a high burden of TB have ignored undernutrition monitoring and treatment as TB control standards, thus addressing undernutrition in these areas should be prioritized to develop and implement actions toward, no poverty, zero hunger, good health, and well-being (UN, 2021).

## The synergistic burden of undernourishment, tuberculosis and COVID-19 pandemic

Worldwide, undernutrition has been considered as the secondary immunodeficiency leading cause, it weakens the adaptive and innate immune system to TB (Morales et al., 2023). Studies have shown that undernutrition increases TB incidence and mortality among adults as well as children (Justine et al., 2020). Nowadays, among the leading risk factors for TB, undernutrition accounts for 15% of the population-attributable fraction (PAF), followed by 7.6 and 3.1% PAF for HIV and diabetes respectively (WHO, 2022b). The considerable limitation of studies of TB and undernutrition resides in the bidirectional interaction between these two conditions since losing weight is a major TB symptom. Therefore, undernutrition would be a TB consequence and not a leading cause of it. However, studies showed that an increase in Body Mass Index (BMI) of 1 kg/m^2^, contributed to a decrease of 13.8% in TB incidence. Similarly, TB incidence increases from 24.7 to 260.2 per 100,000 person-years by the decrease of BMI from normal to less than 18.5 kg/m^2^. Furthermore, when it comes to micronutrients, studies revealed a strong association between vitamin A, E, and D deficiencies and a 2 to 10 times greater increase in progression from latent to active TB (Carwile et al., 2022). Most importantly, COVID-19 pandemic have worsened food insecurity and malnutrition in many low- and middle-income countries (Al Khatib, 2024), the burden of malnutrition increased TB cases due to weaken immune system with sever COVID-19 outcomes and vice versa, which creates a vicious cycle in low resources setting with synergistic burden on healthcare systems.

## Tuberculosis incidence and mortality during COVID-19 pandemic

The impact of tuberculosis (TB) on COVID-19 severity and mortality is unclear, unlike other comorbidities like COPD, cerebrovascular disease, hypertension, diabetes, and cardiovascular disease (WHO, 2022a). A meta-analysis of 6 Chinese studies found that TB was not associated with increased mortality in COVID-19 patients but with a 2.10-fold increase in severe disease progression (Zheng et al., 2020). An Italian observational retrospective study found no significant clinical deterioration in TB/COVID-19 cases (Tadolini et al., 2020). Another study found a high prevalence of TB-associated risk factors and low rates of severe or complicated TB presentation (Canetti et al., 2022).

**Figure 1:**
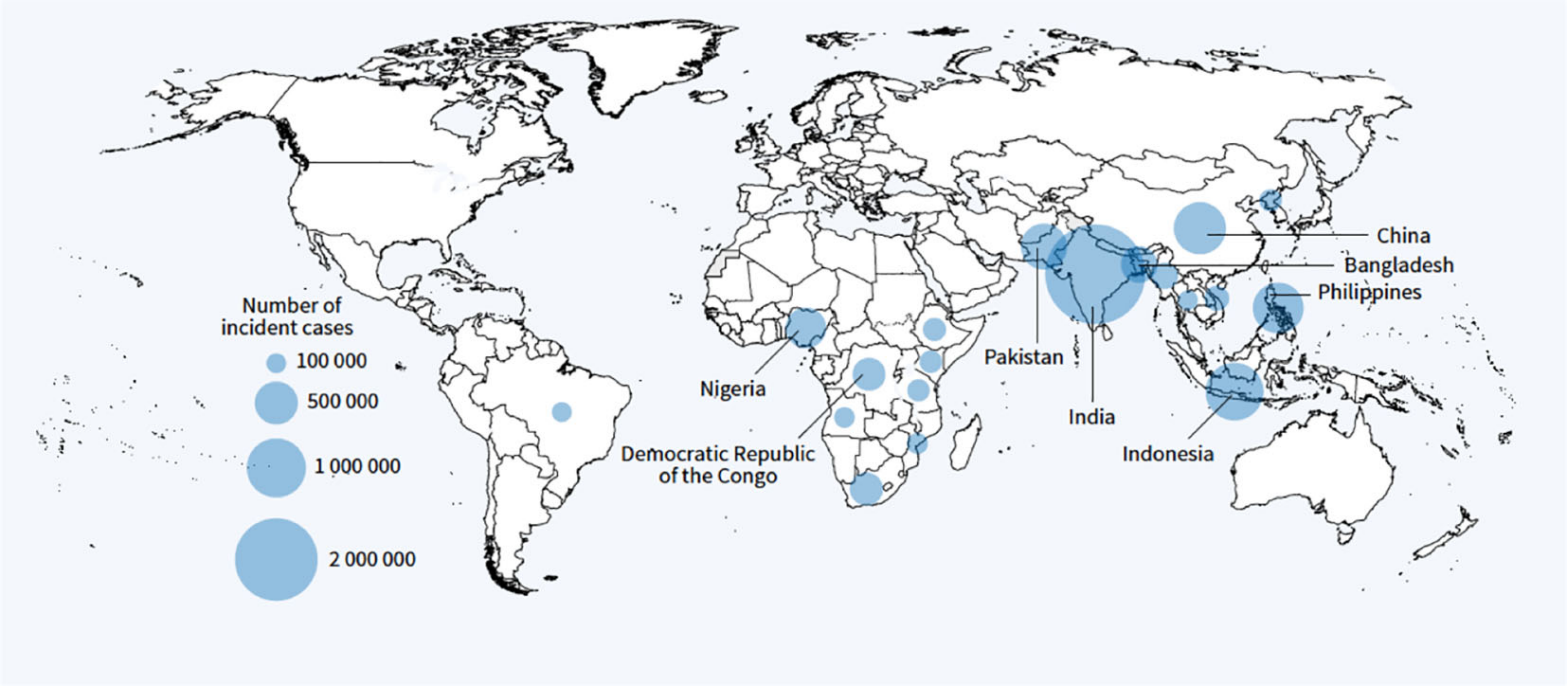
Estimated TB incidence in 2021, for countries with at least 100 000 incident cases. The countries that rank first to eight in terms of number of cases, and that accounted for about two birds of global cases in 2021, are labelled. Source: (WHO, 2022a). Available from: https://www.who.int/teams/global-tuberculosis-programme/tb-reports/global-tuberculosis-report-2022.

**Figure 2:**
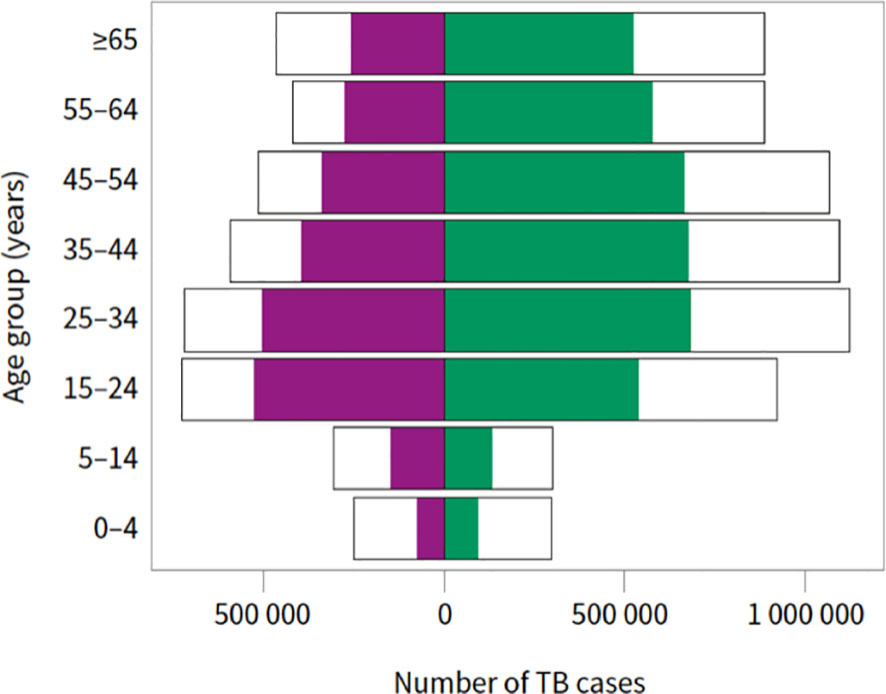
Global estimates of TB incidence (blank outline) and case notifications of people newly diagnosed with TB disaggregated by age and sex (female in purple; male in green), 2021. Source: (WHO, 2022b). Available from: https://www.who.int/teams/global-tuberculosis-programme/tb-reports/global-tuberculosis-report-2022.

Epidemiologically, the incidence rate of a disease is the number of new cases in a given interval of time over the total population at risk during the same period of time. Therefore, the incidence rate of a disease reflects how swiftly the disease occurs within a population. Regarding tuberculosis, the slow decline in the global TB incidence rate was reversed by an increase between 2020 and 2021 (Chakaya et al., 2022). Regionally, the impact of COVID-19 differs from one country to another, thus out of six WHO regions, five countries have shown an increase in TB incidence rate during 2020-2021 (Caren et al., 2022). Among all estimated global TB incidence cases, about 87% are shared by the 30 high TB burden countries where India, Indonesia, China, the Philippines, Pakistan, Nigeria, Bangladesh, and the Democratic Republic of the Congo are ranked as the top 8 countries (**Figure 1**) (WHO, 2022b). Accordingly, TB is more common in developing countries where people live in poverty, in overcrowded places, and work in unhealthy occupation conditions. Moreover, TB infectious agent is more likely to spread in urbanized cities with high levels of water, soil, and air pollution. The target of TB elimination is almost achieved in some WHO Region of the Americas and the European Region and a few countries in the WHO Eastern Mediterranean and Western Pacific regions, where the lowest TB incidence rate was recorded. For the high TB burden countries, the incidence rate of the disease increases remarkably to 150 400 cases per 100,000 population yearly. Similarly, the TB mortality trend has reversed in 2021 to achieve the level of 2017, where the number of TB deaths increased from 1.4 million in 2019 to 1.6 million deaths where HIV-negative people accounted for 1.4 million deaths (WHO, 2022a). Globally, as reported by WHO, the population aged between 25 and 54 besides the male gender bear more burden of the disease (**Figure 2**), where adult men, women, and children accounted for 56.5, 32.5, and 11% of cases respectively. The non-medical or lifestyle factors are among the risk factors that render men more susceptible for developing TB than women (Caren et al., 2022).

During the COVID-19 pandemic, many diseases faced limited access to healthcare services due to limited resources. Tuberculosis (TB) has been one of the most penalized diseases, with significant delays in diagnosis and treatment, leading to increased morbidity and mortality. Both COVID-19 and TB share similar pathogenetic pathways, affecting the lungs (Sanduzzi Zamparelli et al., 2022). However, data on the impact of TB on COVID-19 severity and mortality is unclear, and literature reports are often conflicting. In recent decades, there have been significant shifts in the epidemiology of TB, characterized by a gradual decrease of approximately one-third in both the number of new cases and the death rate associated with the disease (WHO, 2021). However, the COVID-19 pandemic has abruptly reversed this trend, with long-lasting lockdowns, restrictions, and reduced access to healthcare resulting in a drop in access to tuberculosis health services. This reduction in tuberculosis testing is confirmed by the latest global tuberculosis report by the WHO, showing a decline of 18% in TB case notifications in 2020 compared to the previous year (WHO, 2021).

## Mitigating the impact of the COVID-19 pandemic on TB services

In the study “The potential impact of the COVID-19 pandemic on the tuberculosis epidemic a modelling analysis” conducted by Cilloni and his colleagues, it was proved that any interference in TB care results on an long-term adverse impact by TB increasing incidence and mortality rates. Additionally, the lockdown during the pandemic have decreased up to 50% the transmission rate of TB but this decrease was temporary, since disruption of TB services for several months would result, by projection over the 5-6 coming years, in an increase in 119 million TB cases and 361,000 TB deaths in India, 24,700 TB cases and 12,500 deaths in Kenya, and 4,350 cases and 1,340 deaths in Ukraine. The accumulation of undetected TB cases during lockdown is the major driver of this adverse impact of the pandemic on TB incidence and mortality. However, this long-term change on TB could is reversible through intensive detection and treatment of TB cases (Cilloni et al., 2020). These findings were in accordance with those documented in the consolidation report issued by WHO in 2022 which included six case studies conducted between August 2021 and February 2022. The report focused on the implementation of bidirectional screening for TB and COVID-19, supporting real-time surveillance to improve detection of TB, COVID-19 vaccination programs, digital technologies to support treatment adherence and reduce health facility visits, and some case studies addressed socioeconomic determinants or the consequences of TB. In this context of care cycle around 50% of the case studies declared that TB patients showed an improvement in treatment initiation and compliance. The dual screening of TB and SARS-CoV-2 resulted in a surprising decrease in new cases of TB during the pandemic (WHO, 2022a). Therefore, the implementation of successful and feasible action plans such as early detection, initiation and adherence to treatment and reporting new TB cases using digital technologies is crucial to reverse the adverse effect during the pandemic and any waves in the future.

## System approach to control tuberculosis

Countries with a high burden of TB require a complex approach of multisectoral collaboration for disease control and prevention. At the national level, the approach should ensure that guidelines for infection prevention and control of TB are elaborated and updated by the policymakers and public health officials and that the government is providing centralized TB healthcare services. Health facilities and clinics could be the source of the spread of TB within this population (Kuyinu et al., 2016), thus the suggested system approach will address the infrastructure and resources to insist that all TB measures are implemented at the meso level. On the other hand, human behaviors are a great issue that should be tackled at the meso level, since population awareness (screening and adherence to TB treatment) and behavior (personal hygiene, cough etiquette, good housing) greatly affect the disease trend within and across the population (Madebo et al., 2023).

## Challenges to the global control of TB

Unidentified TB cases, financial support, and TB stigma are the greatest challenges that may affect the implementation of this approach. Therefore, targeted screening should be performed for individuals at high risk of developing TB infection, increasing funds to ensure accessibility to treatment and good health services, and increasing awareness within the population toward the disease. All these measures should be prioritized to overcome inequalities and disparities to fight the TB epidemic. Accordingly, the effectiveness of this approach resides in the control of TB through the epidemiological triad (Guo et al., 2019), where the control and prevention of communicable diseases, require a good understanding of its chain of infection and how the infectious agent, host, and environment are interacting to implement the appropriate action at the right point.

## Conclusion

The reduction of the burden of TB at the regional level is a national mission to prioritize and address factors that relatively contribute to the spread of the disease. Accordingly, the decline in the number of TB cases and deaths in some parts of the world was strongly correlated with the improvement of nutrition and housing, income increase, development of health care services, health coverage, and anti-TB drug availability as well as population adherence to treatment regimen (WHO, 2023). Moreover, one of many lessons taught during the pandemic is that any increase in the burden of TB due to lockdown-associated healthcare interference can be addressed when restrictions are lifted, when TB services are restored and targeted interventions are implemented such as the use of digital technologies and dual screening of TB and SARS-CoV-2.
